# *GSK-3β* Regulates Tumor Stemness and Immune-Related Pathways in Triple-Negative Breast Cancer: A Bioinformatics and Experimental Validation Study

**DOI:** 10.1155/tbj/5943807

**Published:** 2025-11-20

**Authors:** Ling Zhou, Xinyu Wang, Huiyin Zhu, Yang Chen, Lifen Bai, Chunyu Hu, Yuhan Wang, Mengfan Qi, Lu Yin, Jian Sun

**Affiliations:** ^1^Department of Thyroid and Breast Surgery, Shanghai Fourth People's Hospital, School of Medicine, Tongji University, Shanghai 200434, China; ^2^Abdominal Surgery Difficult Diagnosis and Treatment Center, Shanghai Tenth People's Hospital, School of Medicine, Tongji University, Shanghai 200072, China

**Keywords:** bioinformatics, *GSK-3β*, triple-negative breast cancer, tumor stemness

## Abstract

**Objective:**

Given the crucial roles of *GSK-3β* in epithelial–mesenchymal transition (EMT), we assume that it may also be involved in tumor stemness, immune evasion, and drug resistance in triple-negative breast cancer (TNBC). This study was designed to analyze the expression and clinical significance of *GSK-3β* and investigate its association with tumor stemness–related and immune-related genes.

**Methods:**

*GSK-3β* expression and clinical data of TNBC patients were obtained from TCGA. Survival analysis, differential gene expression, and gene set enrichment analysis (GSEA) were performed to explore associations between *GSK-3β* and tumor stemness, immune response, and clinical outcomes in TNBC. Immune cell infiltration was assessed using xCell, and key *GSK-3β*-related proteins were validated via parallel reaction monitoring–based proteomics.

**Results:**

*GSK-3β* expression was significantly upregulated in TNBC and was associated with poorer overall survival. In TNBC, 24 *GSK-3β*-associated genes linked to tumor stemness and immune response were identified, all of which were downregulated in the high *GSK-3β* expression group. Proteomic analysis further validated differential expression of key proteins, including upregulation of SERPINB2, KIT, and NOTCH1 and downregulation of DNMT1, MAPK1, and EP300 in *GSK-3β*-overexpressing cells.

**Conclusion:**

*GSK-3β* overexpression was associated with poor prognosis and was found to influence tumor stemness, immune modulation, and key signaling pathways that drive tumor progression and therapeutic resistance.

## 1. Introduction

Triple-negative breast cancer (TNBC) is an aggressive form of breast cancer (BC) characterized by the absence of estrogen receptor (ER), progesterone receptor (PR), and human epidermal growth factor receptor 2 (HER2) expression [1]. Despite recent advances in immunotherapy and antibody–drug conjugates, treatment outcomes remain unsatisfactory, with patients facing poor prognosis, high recurrence rates, and limited targeted therapeutic options [2].

Cancer stem cells (CSCs) exhibit high resistance to therapy, along with enhanced metastatic potential and immune evasion [3]. Although chemotherapy and radiotherapy can eliminate the bulk of non-CSC tumor cells, CSCs often persist and drive disease recurrence and distant metastasis [4]. Despite considerable progress, identifying key molecular regulators of TNBC progression, immune escape, and therapy resistance remains critical for developing more effective therapeutic strategies.

Glycogen synthase kinase-3 beta (*GSK-3β*) is a multifunctional serine/threonine kinase involved in various cellular processes, including proliferation, apoptosis, differentiation, and epithelial–mesenchymal transition (EMT) [5, 6]. During EMT, cells may enter a quiescent state, ceasing division and becoming more resistant to conventional therapies, which primarily target actively dividing cells [2, 7]. The activation of EMT can enhance CSC properties and induce a dormant state, contributing to therapeutic resistance. Given that *GSK-3β* plays a role in EMT regulation, it is plausible that it also influences tumor stemness, immune evasion, and drug resistance. However, the precise mechanisms and target genes or proteins in TNBC remain unclear.

In this study, we first analyzed the expression and clinical significance of *GSK-3β* in TNBC using data from the Cancer Genome Atlas (TCGA). We then investigated its association with tumor stemness–related and immune-related genes and performed gene set enrichment analysis (GSEA) to identify key pathways regulated by *GSK-3β*.

## 2. Materials and Methods

### 2.1. Data Source

*GSK-3β* expression and clinical data from 1118 BC samples and 113 normal samples were obtained from the TCGA database (https://portal.gdc.cancer.gov/repository). We excluded samples lacking tumor and node data, as well as male samples. Finally, 1005 BC samples were included, comprising 108 TNBC samples, 33 ER- and PR-negative but HER2-positive samples, 343 ER- and PR-positive but HER2-negative samples, and 87 samples positive for ER, PR, and HER2.

The UK–Canada METABRIC cohort, consisting of 2509 BC samples with gene expression and clinical data, was used as the validation set. After excluding samples with missing ER, PR, or HER2 status, as well as samples with a survival time of 0, 1979 samples remained, including 319 TNBC cases. Detailed information is provided in Table 1.

### 2.2. Overall Survival (OS) Analysis in the BC and TNBC Patients With Different *GSK-3β* Expression

The patients were stratified based on the optimal cutoff value of *GSK-3β* expression. Then, prognostic significance of *GSK-3β* expression in BC/TNBC was analyzed using the R packages “survival” and “survminer,” with OS as the outcome measure. The prognostic value of *GSK-3β* was validated using the METABRIC dataset.

### 2.3. Identification of *GSK-3β*-Associated Tumor Stemness- and Immune-Related Genes

To identify differentially expressed genes (DEGs) associated with *GSK-3β*, the 108 TNBC samples were divided into high and low *GSK-3β* expression groups based on the median expression value. DEGs were identified using the R package “limma,” with selection criteria of *p* value < 0.05 and |logFC| > 1.5. Next, CSC-related genes were retrieved using the search term “Cancer Stem Cell TNBC *Homo sapiens* (human)” from the National Center for Biotechnology Information Gene database (https://www.ncbi.nlm.nih.gov/gene). Immune-related genes were retrieved from the GeneCards database (https://www.genecards.org/) by querying “Immunologically relevant genes human.” Genes with a relevance score of greater than 12 were selected for subsequent analysis. The intersection of DEGs, CSC-related genes, and immune-related genes were then identified as the subset of *GSK-3β*-associated genes implicated in both tumor stemness and immune regulation.

### 2.4. GSEA Analysis

To explore the functional landscape associated with *GSK-3β* in TNBC, genome-wide Spearman correlation analysis was performed to assess the association between *GSK-3β* expression and the expression of 19,938 genes in TNBC samples. The generated ranked gene list was subjected to GSEA using the R packages “clusterProfiler” and “org.Hs.eg.db,” with gene sets obtained from Molecular Signatures Database (MSigDB) (https://www.gsea-msigdb.org/gsea/msigdb/index.jsp), including “c2.all.v2024.1.Hs.entrez.gmt” and “c5.all.v2024.1.Hs.entrez.gmt.” Enrichment was considered significant when the absolute normalized enrichment score |(NES)| exceeded 1, with a nominal *p* value < 0.05 and a false discovery rate (FDR) *q* value < 0.25.

### 2.5. Immune Infiltration Analysis

xCell, a computational method that estimates the abundance of 64 immune and stromal cell types such as T cells, B cells, macrophages, and fibroblasts [8], was applied to 108 TNBC samples to assess immune cell infiltration. Correlations between the expression of intersecting genes and immune cell abundance were calculated using the R package psych and visualized in a correlation matrix.

### 2.6. Parallel Reaction Monitoring (PRM)–Based Proteomic Analysis

To validate the *GSK-3β*-associated genes identified through bioinformatics analysis, PRM-based proteomic analysis was performed. Negative control (NC) and *GSK-3β*-overexpressing (OE) MDA-MB-231 cells (RRID: CVCL_0062) were seeded in 9 cm culture dishes. Upon reaching a confluence of approximately 80%, cells were washed gently with prechilled phosphate-buffered saline (PBS) at 4°C under slow agitation. Cells were then harvested using a cell scraper, transferred into centrifuge tubes, and centrifuged at 1000 rpm for 10 min. Target proteins and corresponding peptides were selected from the DPHL2.0 spectral library, which contains spectral data for over 14,000 proteins derived from 24 human tissue types [9]. Peptides were extracted from each cell precipitate, followed by desalting using SOLAμTM SPE Plate (Thermo Fisher Scientific) according to the user guides provided by the producer. Afterward, liquid chromatography-tandem mass spectrometry (LC-MS/MS) analysis was performed with the UltiMate 3000 RSLCnano Liquid Chromatography System coupled to an Orbitrap ExplorisTM 480 mass spectrometer (Thermo Fisher Scientific). Data were analyzed using Skyline software (Version 22.2.0.527), and differentially expressed proteins were identified based on an adjusted *p* value < 0.05.

### 2.7. Statistical Analysis

All data were processed and analyzed using R software (v.4.4.2) and GraphPad Prism (v.9.3.1). Comparisons between groups for continuous variables were conducted using the independent sample *t*-test. All statistical tests were two-tailed, with a *p* value < 0.05 considered statistically significant.

## 3. Results

### 3.1. Expression of *GSK-3β* in BC and TNBC

**Figure 1:**
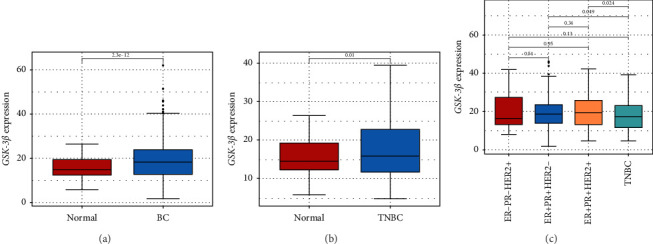
Expression of *GSK-3β* in BC (a), TNBC (b), and other three types of BC (ER−, PR−, HER2+; ER+, PR+, HER2−; ER+, PR+, HER2+) (c).

TCGA database analysis revealed that *GSK-3β* was significantly upregulated in both BC and TNBC compared to the control group (all *p* < 0.05, Figures 1(a) and 1(b)). Moreover, *GSK-3β* expression in TNBC exhibited a statistically significant difference compared to its expression in the TNBC and ER + PR + HER2-subtypes (all *p* < 0.05, Figure 1(c)).

### 3.2. High *GSK-3β* Expression Was Associated With Poorer Prognosis in BC and TNBC

**Figure 2:**
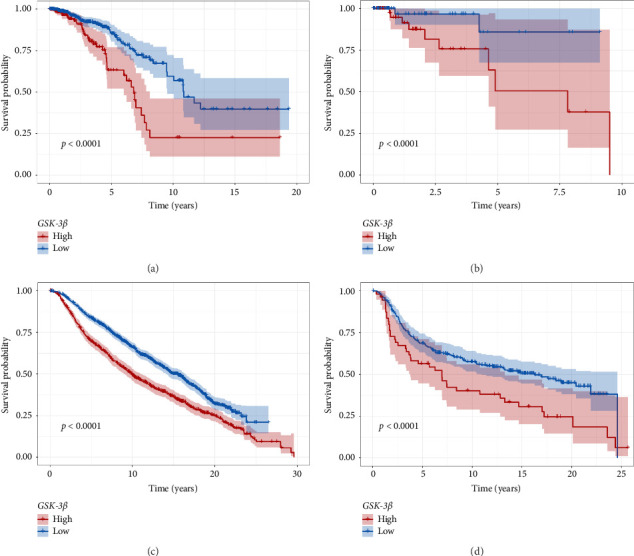
Overall survival of BC (a) and TNBC (b) in *GSK-3β* high- and low-expression groups. Overall survival of BC (c) and TNBC (d) in high- and low- *GSK-3β* expression groups in the validation set.

Based on the optimal cutoff value of *GSK-3β* expression (24.0126), 1005 BC samples were categorized into high-expression group (*n* = 256) and low-expression group (*n* = 749). The OS was significantly shorter in the high-expression group compared to the low-expression group (*p* < 0.05, Figure 2(a)). Similarly, using the optimal cutoff value (15.5641) of *GSK-3β* expression, 108 TNBC samples were divided into high-expression group (*n* = 57) and low-expression group (*n* = 51). Prognostic analysis revealed that the high-expression group exhibited a significantly shorter OS compared to the low-expression group (*p* < 0.05, Figure 2(b)). In the validation cohort, the 1979 BC samples were divided into a high *GSK-3β* expression group (*n* = 800) and a low expression group (*n* = 1179) using the optimal cutoff value of 0.15. Prognostic analysis indicated that patients with high *GSK-3β* expression had shorter OS (Figure 2(c)). Similarly, the 319 TNBC samples were stratified into a high expression group (*n* = 56) and a low expression group (*n* = 263) based on the optimal cutoff value of 1.26, and high *GSK-3β* expression was also associated with shorter OS in TNBC (Figure 2(d)).

### 3.3. GSEA Analysis for *GSK-3β* in TNBC

**Figure 3:**
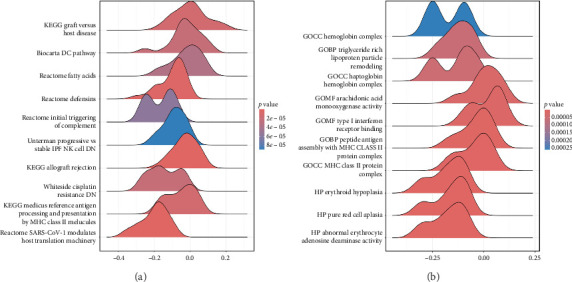
Gene set enrichment analysis ridge plots of *GSK-3β* in TNBC based on C2 (curated gene) (a) and C5 (ontology gene) (b) sets in MSigDB.

The GSEA analysis of *GSK-3β* in the C2 and C5 sets of MSigDB is shown in Figures 3(a) and 3(b), with the top 10 enrichment results sorted based on the |NES| value. The enrichment results in C2 sets showed that *GSK-3β* may be related to SARS-CoV-1–modulated host translation machinery, antigen processing and presentation by MHC class II molecules, genes downregulated during cisplatin resistance, allograft rejection, downregulated genes in NK cells between progressive and stable idiopathic pulmonary fibrosis, initial triggering of complement, defensins, fatty acids, and DC pathways.

The enrichment results in C5 sets revealed that *GSK-3β* may be related to abnormal erythrocyte adenosine deaminase activity, pure red cell aplasia, and erythroid hypoplasia. Furthermore, the Gene Ontology (GO) analysis for *GSK-3β* showed that cellular components (CCs) were enriched in MHC class II protein complex, hemoglobin complex, and haptoglobin–hemoglobin complex. Biological processes (BPs) were enriched in peptide antigen assembly with MHC class II protein complex and triglyceride rich lipoprotein particle remodeling. Molecular functions (MFs) were enriched in type I interferon receptor binding and arachidonic acid monooxygenase activity.

### 3.4. *GSK-3β*-Associated Tumor Stemness- and Immune-Related Genes in TNBC

**Figure 4:**
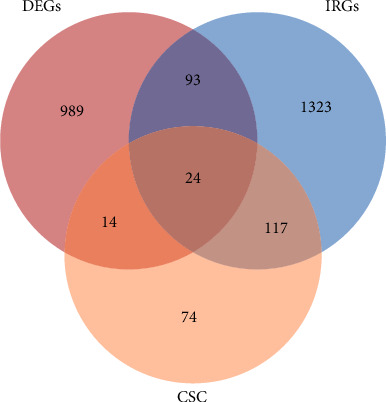
Venn diagram illustrates the differentially expressed genes between the high- and low-*GSK-3β* expression groups, as well as cancer stem cell–related and immune-related genes associated with *GSK-3β*, along with the overlapping genes.

A total of 24 overlapping genes were identified among the 1120 DEGs, 229 CSC-related genes, and 1557 immune-related genes associated with *GSK-3β* (Figure 4). These overlapping genes included *TGFA*, *FOXO3*, *BRCA2*, *NOTCH1*, *ITGB1*, *GSK3β*, *CTNNB1*, *KDR*, *MAPK14*, *EGFR*, *EP300*, *SMC1A*, *ADAM17*, *MAPK1*, *TOP2A*, *MUC1*, *MTOR*, *PTPN11*, *KIT*, *NTRK2*, *MKI67*, *IGF1R*, *DNMT1*, and *ARID1A*. Compared to the low *GSK-3β* expression group, all these genes were significantly downregulated in the high-expression group (*p* < 0.05, Table S1).

### 3.5. Proteomic Analysis–Validated *GSK-3β*-Associated Proteins in TNBC

**Figure 5:**
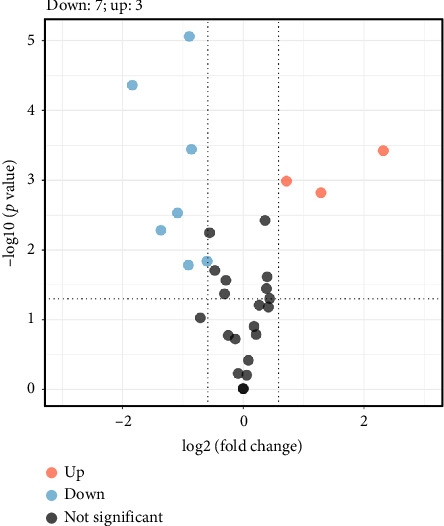
Differentially expressed proteins in normal and *GSK-3β*-overexpression MDA-MB-231 cells.

Based on the bioinformatics analysis results, we performed proteomic analysis to investigate the expression of 30 proteins in normal and *GSK-3β*-overexpression MDA-MB-231 cells (Figure S1). Compared with the normal control, SERPINB2, KIT, and NOTCH1 were significantly upregulated in the *GSK-3β*-overexpression group, whereas COX2, DNMT1, MAPK1, AKT1, MK167, EP300, and SMC1A were significantly downregulated (*p* < 0.05, Figure 5 and Table S2).

## 4. Discussion

TNBC is an aggressive subtype of BC with limited targeted treatment options and poor prognosis [1]. Increasing evidence indicates that dysregulated signaling pathways and the tumor immune microenvironment play key roles in its progression [10], yet the prognostic and therapeutic value of specific regulators such as *GSK-3β* remains unclear. In this study, we demonstrated that *GSK-3β* is significantly associated with the clinical outcomes in TNBC patients. Elevated *GSK-3β* expression was correlated with poor prognosis, suggesting its potential role as a prognostic biomarker. Further analysis revealed that *GSK-3β* was linked to tumor stemness- and immune-related pathways. Enrichment of pathways suggests that *GSK-3β* may promote TNBC progression by modulating both tumor-intrinsic and microenvironmental mechanisms. Our immune infiltration analysis further highlights the immunomodulatory role of *GSK-3β*, suggesting its potential as a therapeutic target in combination with immune checkpoint inhibitors. To experimentally validate the bioinformatic findings, we conducted PRM-based proteomic profiling of 30 selected proteins in normal and *GSK-3β*-OE MDA-MB-231 cells. Compared with control cells, SERPINB2, KIT, and NOTCH1 were significantly upregulated upon *GSK-3β* overexpression, while COX2, DNMT1, MAPK1, AKT1, MKI67, EP300, and SMC1A were markedly downregulated.

NOTCH1, a key regulator of self-renewal and differentiation in CSCs, contributes to therapeutic resistance in TNBC [11]. Its upregulation indicates a potential role in enhancing the stemness properties of TNBC cells, thereby increasing tumor aggressiveness and relapse risk [12]. As a receptor tyrosine kinase, KIT has been implicated in the maintenance of stem-like properties and resistance to chemotherapy in TNBC [13]. The observed increase in KIT expression upon *GSK-3β* overexpression suggests that this pathway may promote a more aggressive, therapy-resistant phenotype by reinforcing stem-like characteristics. This dual effect on stemness and therapeutic resistance highlights *GSK-3β* as a promising target for novel combination therapies aimed at eradicating resistant tumor subpopulations in TNBC [14, 15].

The downregulation of AKT1 and MAPK1 in the presence of *GSK-3β* overexpression indicates that it may reprogram cellular signaling away from a proliferative phenotype. AKT1 is a crucial downstream effector of the PI3K/AKT pathway, which promotes cell survival, growth, and metabolism [16], while MAPK1 is central to the MAPK/ERK signaling cascade, which regulates cell proliferation, differentiation, and survival [17]. The suppression of these pathways suggests that *GSK-3β* overexpression may induce a shift from an actively proliferating state to a more quiescent or stem-like state. This could have profound implications for tumor progression, as tumor cells that adopt a stem-like phenotype are typically more resistant to conventional chemotherapy and radiation treatments [18]. Moreover, the modulation of these pathways may contribute to the altered proliferation observed in *GSK-3β*-OE cells, suggesting that *GSK-3β*-mediated reprogramming provides a mechanism for TNBC cells to survive and persist under therapeutic pressure.

The downregulation of DNMT1 and EP300 provides further evidence that *GSK-3β* may play a role in epigenetic reprogramming in TNBC. DNMT1 is responsible for maintaining DNA methylation patterns during cell division, and its downregulation could lead to the reactivation of tumor suppressor genes that were previously silenced by hypermethylation [19]. This reactivation could shift the balance of gene expression toward a more differentiated or less aggressive phenotype, although the exact outcome would depend on the specific genes involved [20]. Similarly, EP300, a histone acetyltransferase, is responsible for modifying chromatin structure and regulating gene expression [21]. The downregulation of EP300 in response to *GSK-3β* overexpression suggests that *GSK-3β* may remodel the chromatin landscape, thereby altering gene expression to promote a more aggressive, stem-like phenotype. These findings suggest that *GSK-3β* may modulate both DNA methylation and histone acetylation, contributing to a broader epigenetic reprogramming of TNBC cells.

As a key enzyme in inflammation and immune response, the downregulation of COX2 offers additional insight into the role of *GSK-3β* in shaping the tumor microenvironment. COX2 is typically upregulated in many cancers, which plays a key role in immune evasion by promoting the production of proinflammatory mediators [22]. Its downregulation in *GSK-3β*-OE cells suggests that *GSK-3β* may suppress inflammation, potentially creating an immune-suppressive niche that favors tumor survival and progression [23]. This may facilitate the evasion of immune surveillance, indicating that *GSK-3β* functions in both tumor progression and immune escape. By downregulating COX2, it may reshape the tumor microenvironment, thereby enhancing the resistance of TNBC cells to immune-mediated destruction and ultimately contributing to the poor prognosis.

In this study, several limitations should be addressed. First, our proteomic analysis was limited to a single cell line, requiring validation in additional models. Second, the epigenetic mechanisms underlying *GSK-3β* modulation remain unclear and further investigation is still required. Third, larger clinical cohorts and long-term follow-up studies are necessary to validate *GSK-3β* as a prognostic biomarker and therapeutic target.

## 5. Conclusions

Our findings demonstrate that *GSK-3β* overexpression was associated with poor prognosis. By regulating tumor stemness, immune modulation, and critical signaling pathways, *GSK-3β* contributes to tumor progression and therapeutic resistance. Proteomic profiling further revealed its impact on multiple oncogenic and tumor-suppressive proteins, with notable upregulation of SERPINB2, KIT, and NOTCH1, alongside downregulation of COX2, DNMT1, MAPK1, AKT1, MKI67, EP300, and SMC1A. These results suggest that *GSK-3β* may be a promising target for novel combination therapies, particularly when combined with immune checkpoint inhibitors, to enhance treatment efficacy in TNBC patients.

## Figures and Tables

**Table 1:** Data source.

Database	Accession/name	Sample size (*n*)
TCGA	TCGA-BRCA	113 normal, 1118 BC
UK–Canada METABRIC project	Breast Cancer (METABRIC, Nature 2012 & Nat Commun 2016)	2509 BC

## Data Availability

All data generated or analyzed during this study are included in this published article and its supporting information files.

## Supporting Information

Supporting Information 1 Figure S1: validation of *GSK-3β* overexpression in MDA-MB-231 cells. (A) Volcano plot of proteomic analysis shows significant upregulation of *GSK-3β* in *GSK-3β*-overexpressing MDA-MB-231 cells compared to control cells. (B) Western blot analysis of *GSK-3β* overexpression.

Supporting Information 2 Table S1: twenty-four overlapping genes of differentially expressed genes, cancer stem cell-related genes, and immune-related genes associated with *GSK-3β* in TNBC.

Supporting Information 3 Table S2: the expression of 30 proteins in normal (NC) and *GSK-3β*-overexpression MDA-MB-231 cells (NE).
