# Supplementary material for: GSK-3β Regulates Tumor Stemness and Immune-Related Pathways in Triple-Negative Breast Cancer: A Bioinformatics and Experimental Validation Study
Source: Breast J. 2025 Nov 20;2025:5943807. doi: 10.1155/tbj/5943807 (PMC12660618; doi:10.1155/tbj/5943807)
Supplement: Supporting Information 1 — Figure S1: validation of GSK-3β overexpression in MDA-MB-231 cells. (A) Volcano plot of proteomic analysis shows significant upregulation of GSK-3β in GSK-3β-overexpressing MDA-MB-231 cells compared to control cells. (B) Western blot analysis of GSK-3β overexpression. [file 5943807.f1.docx]

**Supplementary Figure**


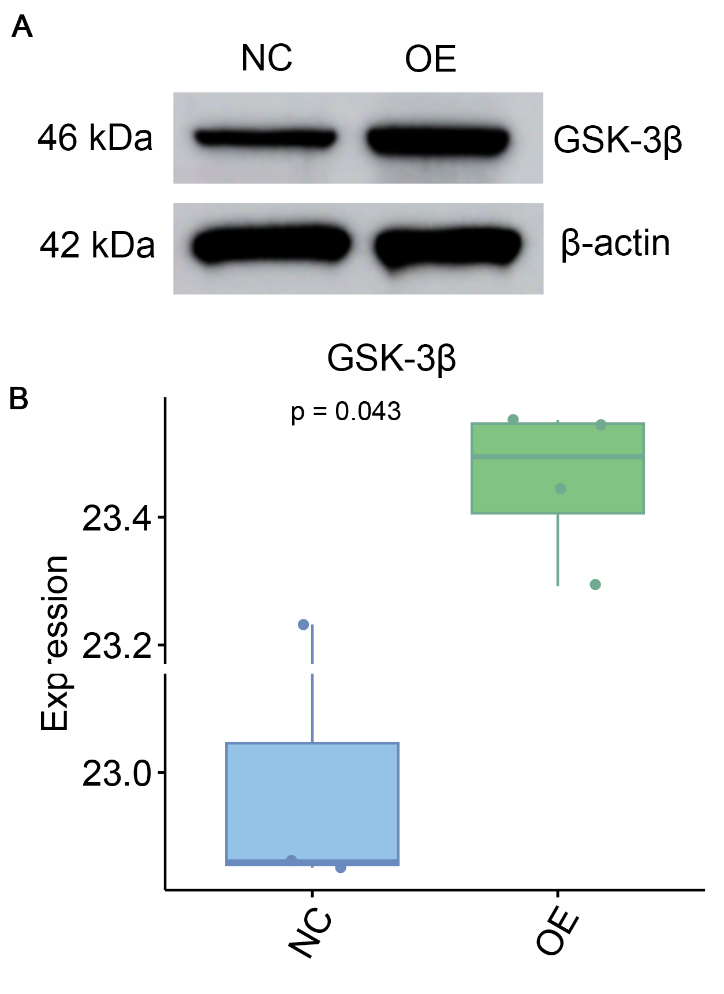


**Figure S1.** Validation of GSK-3β overexpression in MDA-MB-231 cells.

(A) Volcano plot of proteomic analysis shows significant upregulation of GSK-3β in GSK-3β-overexpressing MDA-MB-231 cells compared to control cells.

(B) Western blot analysis of GSK-3β overexpression.
